# Single Step Endodontic Management of Immature Apices using MTA

**DOI:** 10.5005/jp-journals-10005-1122

**Published:** 2011-04-15

**Authors:** RK Tewari, SK Mishra, Ashok Kumar, Amit Kumar Garg, MK Jindal, Harish Juneja

**Affiliations:** 1 Chairman, Department of Conservative Dentistry, Dr ZA Dental College, Aligarh Muslim University, Aligarh, Uttar Pradesh, India; 2 Associate Professor, Department of Conservative Dentistry, Dr ZA Dental College, Aligarh Muslim University, Aligarh, Uttar Pradesh, India; 3 Professor, Department of Conservative Dentistry, Dr ZA Dental College, Aligarh Muslim University, Aligarh, Uttar Pradesh, India; 4 Assistant Professor, Department of Conservative Dentistry, Dr ZA Dental College, Aligarh Muslim University, Aligarh, Uttar Pradesh, India; 5 Associate Professor, Department of Pediatric Dentistry, Dr ZA Dental College, Aligarh Muslim University, Aligarh, Uttar Pradesh, India; 6 Postgraduate Student, Department of Conservative Dentistry, Dr ZA Dental College, Aligarh Muslim University, Aligarh, Uttar Pradesh, India

**Keywords:** Mineral trioxide aggregate, Immature apices, Single step endodontic management.

## Abstract

**Aim:**

To examine the clinical and radiographic appearance of teeth with immature apices that were treated by single step procedure using mineral trioxide aggregate (MTA).

**Summary:**

Creation of a physiological hard tissue barrier with calcium hydroxide in a nonvital tooth although quite predictable has certain limitations, such as the very long duration of the treatment spread over multiple visits and increased risk of root fracture. Plugging the root canal end with MTA has been advocated as an alternative treatment modality for open apices. The technique has been proven to be successful in many recently reported cases. The cases reported here present the successful treatment of two traumatized maxillary central incisors with open apices and periapical lesions using MTA. In this case report, MTA has been used to create a hard tissue barrier after disinfection of the root canal.

## INTRODUCTION

Endodontic management of the pulpless permanent tooth with a wide-open blunderbuss apex has long presented a challenge to the dentistry. Many techniques have been advocated to manage the pulpless immature permanent tooth. The most widely used procedure is the apexification which involves cleaning and filling the canal with a temporary paste (mostly calcium hydroxide) to stimulate the formation of calcified tissue at the apex. The calcium hydroxide technique has its inherent disadvantages, including increased cost; patient compliance with multiple appointments over 12 to 24 months and possible root fracture.^1^ It has been reported that approximately 30% of the teeth will fracture during or after endodontic treatment.^2^

Apical barrier technique using mineral trioxide aggregate (MTA) has recently been advocated as an alternative treatment modality for open apices. MTA induces apical hard tissue formation more often than osteogenic protein-1 or calcium hydroxide while producing less inflammation. MTA is an endodontic material that was developed at Loma Linda University, USA in 1993. It was described for the first time in the dental literature by Lee et al.^3^ Since that time, it has been widely investigated and the results confirm that it has excellent physical properties.

The material was first used as a root-end filling material by Torabinejad et al,^4^ but it has also been used as a viable alternative for various clinical applications, such as capping of pulp tissue, root-end closure and for repairing furcal perforations.^5^ Underlying these applications are the properties of MTA that include biocompatibility, good sealing ability and capability of promoting dental pulp and periradicular tissue regeneration.

MTA might be an ideal material because it consistently induces the regeneration of periodontal ligament tissues, the apposition of a cementum-like material and formation of bone. MTA has been reported to be biocompatible in many *in vivo* and *in vitro* studies. Koh et al^6^ reported that MTA offered a biologically active substrate for bone and cells stimulating interleukin production, and Mitchell et al^7^ reported that MTA was biocompatible and suitable for clinical trials. Zhu et al^8^ reported that osteoblasts have a favorable response to MTA.

MTA is a white or grey powder of fine hydrophilic particles consisting of compounds of tricalcium silicate, tricalcium oxide, tricalcium aluminate and silicate oxide.^2^ Several studies have reported that MTA is similar to commercial Portland cement.^9,10^ It is commercially available as ProRoot MTA (Dentsply Tulsa Dental, Tulsa, OK, USA). Recently, a new cement was launched commercially labelled as MTA-Angelus. This material is composed of 80% Portland cement and 20% bismuth oxide.^11^

## CASE REPORTS

### Case 1

**Fig. 1:**
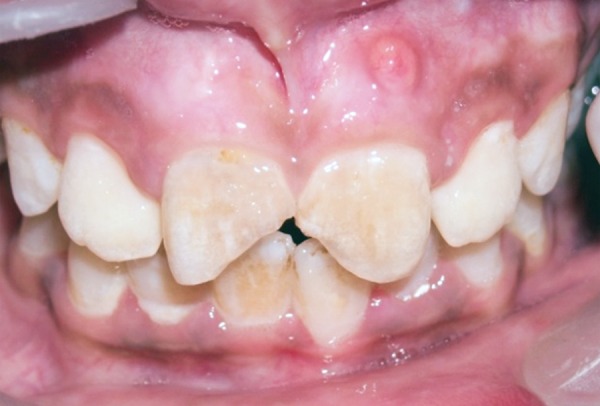
Preoperative photograph showing fractured maxillary central incisors and draining sinus in relation to root of 21

A 12-year-old female patient presented to the Department of Conservative Dentistry and Endodontics of Dr ZA Dental College, with fracture of both maxillary central incisors. There was a history of swelling and pus discharge at regular intervals from the involved teeth. The fracture occurred 3 years back. Medical history was not significant. Clinical examination revealed mesioincisal complicated crown fracture of both teeth with triangular-shaped access opening present on the palatal surface of both the teeth. A sinus was present in relation to the apex of the left central incisor (Fig. 1).

**Fig. 2:**
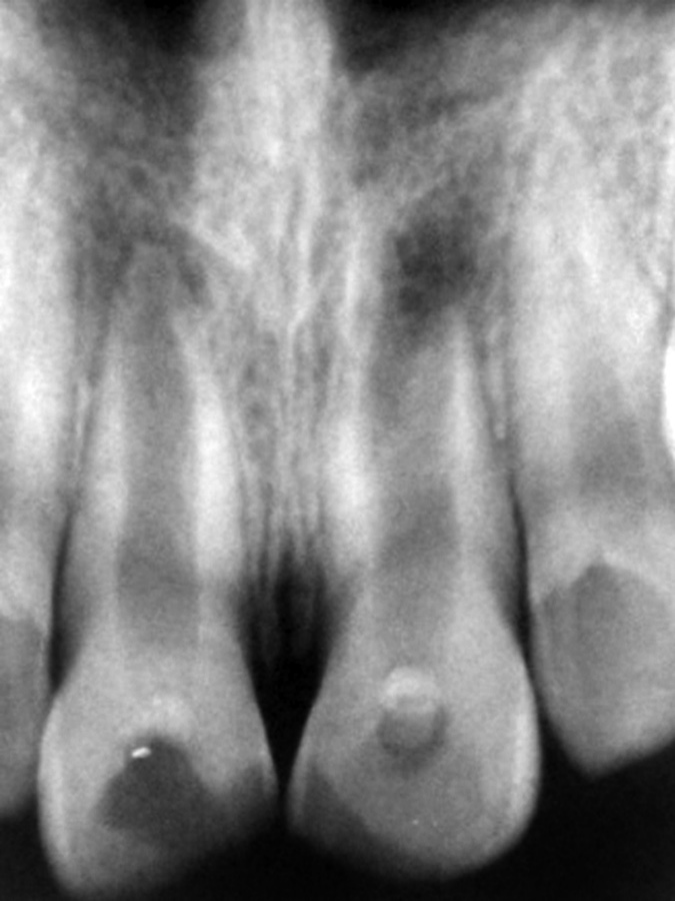
Preoperative radiograph

The patient had earlier reported to a private dental office 2 years back. Treatment with calcium hydroxide was attempted with calcium hydroxide being changed at regular intervals. However, no success was obtained, as apparent from the radiographic examination. Radiographic examination revealed open apices and periapical lesion in relation to apices of both teeth (Fig. 2). Therefore, the new treatment plan was decided to be apical plugging of the orifice with MTA and obturation of the remainder of the canal with gutta-percha.

The teeth were isolated with a rubber dam. A conventional access cavity was earlier prepared which was refined with endo access bur and working length was obtained. The number 80 K-file (Dentsply-Maillefer, USA) was found loose and easily passing beyond the apical limit of the canal. The canal was thoroughly cleaned using instrumentation and 0.5% sodium hypochlorite irrigation. Lower strength of hypochlorite is used because of increased danger of placing NaOCl through the apex of immature teeth. Lower concentration is compensated by the large volume of irrig-ants. The root canals were dried with sterile paper points (Dentsply-Maillefer, USA).

**Figs 3A to C:**
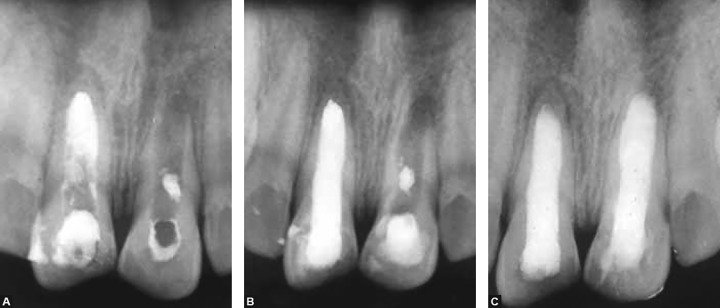
(A) MTA apical plug in tooth 11, (B) after gutta-percha filling of remaining canal in tooth 11 next day and MTA apical filling in tooth 21, (C) final radiograph after gutta-percha filling in tooth 21

The teeth were treated one by one. The white MTA (ProRoot MTA, Dentsply Tulsa Dental, Tulsa, OK, USA) was mixed to a paste consistency with sterile water and delivered to the canal in tooth no.11 using an amalgam carrier in about 4 mm thickness. It was placed to working length with posterior pluggers. Excess of MTA was carefully removed. Sterile sponge pellet moistened with sterile water was placed over the canal orifice and the access cavity was sealed temporarily. Correct placement of MTA was confirmed radiographically. Next day, the tooth was re-opened and hard set of the MTA was verified with an endodontic file. Endoflas root canal sealer (Sanlor and Cia, Cali, Colombia) was applied with a lentulo spiral in a slow-speed handpiece in the remainder of the canal and obturation was done with guttta-percha (Dentsply-Maillefer) and Endoflas sealer using a lateral condensation technique (Figs 3A to C). In the tooth no 21, MTA placement was done at this appointment using the same technique and obturation was completed the next day. At the next appointment, a week later, the teeth were restored with dentine and enamel-bonded composite.

Teeth were reviewed radiographically at the first visit, after performing the apical plug, after filling of the canal and coronal restoration and at every 3-month interval up to 1 year. Healing was evident radiographically, dictated by the decreasing size of the periapical lesions. Clinically, teeth were free from symptoms, buccal sinus tracts and swelling. Radiographic examination revealed almost complete resolution of the periapical lesion.

### Case 2

**Fig. 4:**
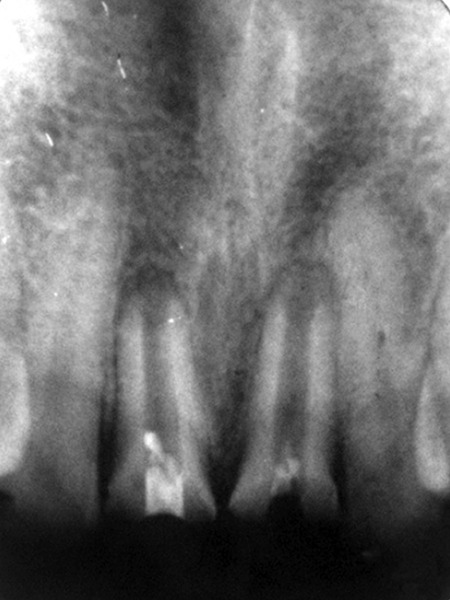
Preoperative radiograph

A 35-year-old male patient presented to the Department of Conservative Dentistry and Endodontics of Dr ZA Dental College, with fracture of both maxillary central incisors. The fracture occurred when the patient was 9-years-old. Patient had the habit of tobacco chewing and both his central incisors were worn down to the gingival level. Radiographic examination revealed open apices in relation to both teeth along with presence of periapical lesions (Fig. 4).

**Figs 5A to C:**
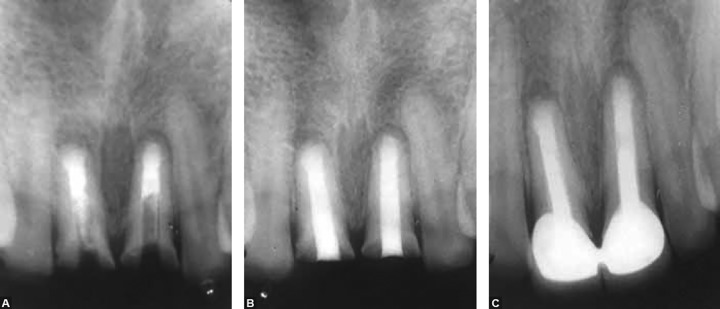
(A) MTA apical filling in 11 and 21, (B) after obturation with gutta-percha in the remainder of the canal the next day, (C) after final prosthetic rehabilitation with cast postcore followed by PFM crowns

Endodontic access was made on the palatal surface of the involved teeth after application of the rubber dam. The same procedure was performed to treat the teeth as in the previous case. Custom-made metal post and core were fabricated and cemented followed by the final prosthetic rehabilitation with the help of porcelain-fused to metal crowns (Figs 5A to C). The patient was followed up at regular intervals up to 1 year; the teeth were free from any clinical symptoms, buccal sinus tracts and swelling. Radiographic examination revealed almost complete healing of the periapical lesion.

## DISCUSSION

The primary objective of nonsurgical root canal therapy in teeth with incomplete root formation is long-term tooth retention. The traditional approach of using calcium hydroxide to facilitate obturation of the root canal space has provided a high degree of success. There are, however, several disadvantages to this treatment modality. Any treatment requiring several visits during a long period of time risks patient attrition as a result of patient fatigue and geographic relocation. If a child moves away during the course of treatment, it is difficult to ensure that the integrity of the coronal seal is maintained, and that treatment is completed.

Likewise, patient compliance can be a problem when multiple visits are necessary. Repeated visits to the dentist can be disruptive and difficult in a busy schedule for both the parent and child. In addition, these appointments are easily forgotten because the patient usually remains asymptomatic, and the tooth looks clinically normal. The long-term application of calcium hydroxide has been shown to weaken the tooth and increase the likelihood of tooth fracture. Thus, a treatment alternative that has a higher rate of long-term success avoids the use of extended applications of calcium hydroxide and minimizes the number of patient visits would be a desirable alternative.

MTA obturation of teeth with open apices avoids many of the problems associated with traditional apexification procedures. MTA is biocompatible and free of inflammation. ^12,13^ It is osteoconductive and promotes osteogenesis when implanted intraosseously.^14^ MTA offers a biologically active substrate for bone cells and stimulates interleukin formation. MTA has also shown to be cementoconducive in tissue cultures with cementoblast attachment to the material and production of mineralized tissue.^15^ MTA is, thus, a viable option and should be considered as a good alternative to routine apexification procedures.

## CONCLUSION

The nonsurgical management ofteeth with necrotic pulps and incomplete apex formation with MTA is a useful treatment option, with many of the advantages when compared with the traditional calcium hydroxide apexification.
